# Prevalence and genetic diversity of *Wolbachia* endosymbiont and mtDNA in Palearctic populations of *Drosophila melanogaster*

**DOI:** 10.1186/s12862-019-1372-9

**Published:** 2019-02-26

**Authors:** Roman А. Bykov, Maria A. Yudina, Nataly E. Gruntenko, Ilya K. Zakharov, Marina A. Voloshina, Elena S. Melashchenko, Maria V. Danilova, Ilia O. Mazunin, Yury Yu. Ilinsky

**Affiliations:** 1 Institute of Cytology and Genetics, Russian Academy of Sciences, Siberian Branch, Novosibirsk, Russia; 2 Novosibirsk State University, Novosibirsk, Russia; 3 Immanuel Kant Baltic Federal University, Kaliningrad, Russia

**Keywords:** *Wolbachia*, *Drosophila melanogaster*, Maternal inheritance, Symbiosis, Mitochondrial DNA, Palearctic

## Abstract

**Background:**

Maternally inherited *Wolbachia* symbionts infect *D. melanogaster* populations worldwide. Infection rates vary greatly. Genetic diversity of *Wolbachia* in *D. melanogaster* can be subdivided into several closely related genotypes coinherited with certain mtDNA lineages. mtDNA haplotypes have the following global distribution pattern: mtDNA clade I is mostly found in North America, II and IV in Africa, III in Europe and Africa, V in Eurasia, VI is global but very rare, and VIII is found in Asia. The wMel *Wolbachia* genotype is predominant in *D. melanogaster* populations. However, according to the hypothesis of global *Wolbachia* replacement, the wMelCS genotype was predominant before the XX century when it was replaced by the wMel genotype. Here we analyse over 1500 fly isolates from the Palearctic region to evaluate the prevalence, genetic diversity and distribution pattrern of the *Wolbachia* symbiont, occurrence of mtDNA variants, and finally to discuss the *Wolbachia* genotype global replacement hypothesis.

**Results:**

All studied Palearctic populations of *D. melanogaster* were infected with *Wolbachia* at a rate of 33–100%. We did not observe any significant correlation between infection rate and longitude or latitude. Five previously reported *Wolbachia* genotypes were found in Palearctic populations with a predominance of the wMel variant. The mtDNA haplotypes of the I_II_III clade and V clade were prevalent in Palearctic populations. To test the recent *Wolbachia* genotype replacement hypothesis, we examined three genomic regions of CS-like genotypes. Low genetic diversity was observed, only two haplotypes of the CS genotypes with a ‘CCG’ variant predominance were found.

**Conclusion:**

The results of our survey of *Wolbachia* infection prevalence and genotype diversity in Palearctic *D. melanogaster* populations confirm previous studies. *Wolbachia* is ubiquitous in the Palearctic region. The wMel genotype is dominant with local occurrence of rare genotypes. Together with variants of the V mtDNA clade, the variants of the ‘III+’ clade are dominant in both infected and uninfected flies of Palearctic populations. Based on our data on *Wolbachia* and mtDNA in different years in some Palearctic localities, we can conclude that flies that survive the winter make the predominant symbiont contribution to the subsequent generation. A comprehensive overview of mtDNA and *Wolbachia* infection of *D. melanogaster* populations worldwide does not support the recent global *Wolbachia* genotype replacement hypothesis. However, we cannot exclude wMelCS genotype rate fluctuations in the past.

**Electronic supplementary material:**

The online version of this article (10.1186/s12862-019-1372-9) contains supplementary material, which is available to authorized users.

## Background

Bacteria of the *Wolbachia* genus are widespread across *Drosophila* species [1, 2]. *Drosophila* spp. are popular model organisms for studies of different aspects of *Wolbachia* biology including microevolution and population dynamics [2–8]. *Wolbachia* can affect the *Drosophila* host in different ways including male killing in *D. bifasciata* and *D. innubila* [9, 10], cytoplasmic incompatibility (CI) in *D. paulistorum*, *D. simulans* and *D. melanogaster* [8, 11, 12], protection from RNA-viruses [13, 14], nutrition provisioning [15], fecundity increasing [16] and suppression of mutations [17–19].

Many *Wolbachia* strains in different *Drosophila* hosts are non-related. There are*,* five genetically distant *Wolbachia* strains in *D. simulans,* which differ in geographical distribution and CI expression [1, 20, 21]. These strains are coinherited with three mtDNA haplotypes (*si*I, *si*II and *si*III) [1, 21, 22], which suggests that at least some of these *Wolbachia* strains have been recently harboured by *D. simulans* [2].

In *D. melanogaster,* the single *Wolbachia* strain, wMel, has been described based on the analysis of some housekeeping genes [23–26]. Studies of *Wolbachia* genomes in different *D. melanogaster* strains have revealed differences in chromosomal rearrangements, variation in *indels* and repetitive sequences [27–31]. These findings allow the subdivision of the wMel strain into six closely related variants (namely genotypes). These variants form two groups; MEL includes wMel, wMel2, wMel3 and wMel4 genotypes, and CS includes wMelCS and wMelCS2 [29, 32]. Further, the whole-genome sequence analysis has revealed several clades of the wMel strain [30, 33, 34]. The MEL genotypes correspond to *Wolbachia* clades I-V and VIII, and the CS genotypes correspond to clade VI [30, 32, 34].

In comparison with *D. simulans* [21, 22], the number of nucleotide polymorphisms in *D. melanogaster* mitochondrial DNA is low. Only analysing *Wolbachia* polymorphisms as genetic marker of maternal inheritance and genome sequence analyses of many *D. melanogaster* isolates have provided valuable data on *D. melanogaster* mtDNA variation. *D. melanogaster* mtDNA and the wMel *Wolbachia* strain are strictly coinherited. mtDNA lineages have been designated as *M*- or *S*-clade according to polymorphisms of some loci [32, 35], and as clades I-VI and VIII according to whole-genome sequencing [30, 33, 34]. As a result, the following associations are observed: *Wolbachia* of MEL group/I-V and VIII clades are associated with mtDNA of *M*/I-V and VIII clades, and *Wolbachia* of the CS group/VI clade are associated with mtDNA of the *S*/VI clade. Phylogenetic analyses revealed the divergence of the wMel *Wolbachia* strain and *D. melanogaster* mtDNA from a common ancestor several thousand years ago [30, 32–34].

There is a global geographic pattern of *D. melanogaster* maternal lineages. The MEL-group genotypes and *M*-clade mtDNA of uninfected flies have been found in *D. melanogaster* populations all over the world [29, 32, 36, 37]. However, this *Wolbachia* group and mtDNA includes lineages of I-V and VIII clades (both mtDNA and *Wolbachia*) that have different geographical distributions. The I and III clades of mtDNA and *Wolbachia* seem to spread across all continents [32–34]. The II and IV clades are found in Africa; the V clade in the Palearctic region, and the VIII clade (associated with wMel2 genotype) in Eastern Asia [30, 32, 33, 38]. The VI clade is associated with CS-group *Wolbachia*. This clade is found all over the world, but its frequency is very low. Previously, an additional mtDNA clade was proposed (clade VII), which is associated with wMelCS2 genotype and distributed in the Palearctic (mainly in Eastern Europe, the Caucasus, Central Asia and South of Western Siberia) [32, 37]. However, Chrostek et al. [30] did not confirm the validity of this clade. Data about diversity and distribution of *D. melanogaster* mtDNA and *Wolbachia* from South America and Australia and many territories in Eurasia are lacking.

There are data on how *Wolbachia* variants of different clades affect *D. melanogaster* environmental adaptation [30, 38–40]. In contrast to *D. simulans*, *Wolbachia* in *D. melanogaster* induce no/low CI or strong CI in the case of ‘young males’ [11, 41, 42]. The reasons for high *Wolbachia* density in *D. melanogaster* populations are unclear because the main factors affecting symbiont spreading are weak, and others, such as protection from viral infections and mutation suppression could only have a localised effect.

In the present study, we evaluated *Wolbachia* and mtDNA prevalence in fly populations across the vast Palearctic region. In particular, we were interested in the frequency and distribution of *Wolbachia* genotypes and mtDNA variants in Palearctic fly populations. We confirmed previous data on widespread *Wolbachia* infection, high infection rates and predominance of the wMel *Wolbachia* genotype in *D. melanogaster* populations, and show 1) low genetic diversity of CS *Wolbachia* genotypes, 2) predominance of two mtDNA clades in Palearctic *D. melanogaster* populations and 3) overwintering flies in urbanized localities. Based on our results, we conclude that global *Wolbachia* genotype replacement has not occurred in the recent past.

## Methods

### Sample collection

Our collection includes 1550 *D. melanogaster* samples from 12 Palearctic regions (43 localities) collected between 1974 and 2015 (Additional file 1). Most of the samples (1505) are from natural populations collected during 2008–2015 and include isofemale lines and alcohol samples. Isofemale lines were analysed in the year of collection to minimize the possibility of stochastic loss of *Wolbachia*. In addition, 45 laboratory isofemale lines from long-term storage, established between 1974 and 2005, were studied for mtDNA polymorphisms. Some were also studied for genetic diversity of CS *Wolbachia* genotypes. Samples were examined for *i*) *Wolbachia* prevalence and genotype diversity (*n* = 1251), *ii*) genetic diversity of CS-genotypes (*n* = 22 including nine long-term storage mutant stocks); *iii*) *M/S* clades of mtDNA (*n* = 1550. Here 254 samples that were studied for *Wolbachia* infection in Bykov et al. [37] were included), and *iv*) I-VIII mtDNA clades (*n* = 143) (Additional file 2).

### Screening and sequencing

DNA extraction was performed according to Ilinsky [32]. *Wolbachia* symbionts in the collection were analysed by PCR using 81F/691R primers for the *wsp* gene [43] and 99F/994R for the *16SrRNA* gene [44]. *Wolbachia* genotypes were determined according to Riegler et al. [29]. According to an analysis of nine complete CS *Wolbachia* genomes from Richardson et al. [33], Chrostek et al. [30] and Versache et al. [38], we found 30 SNP sites among the CS genotypes (Additional file 3). Nineteen sites were non-parsimonious or uncertain and eleven were parsimonious. We chose three parsimonious sites located in coding regions and separated by more than 89 kbp (Additional file 3) to characterize 22 *Wolbachia* isolates with primers WclpBF: 5′-GGCTTTCGCAAGTTCGGTTT-3′, WclpBR: 5′-GGAGAGCTGATGTATGGTGT-3′ (208019–208326 region in *Wolbachia* genome according to GenBank AE017196.1), WlonF: 5′-CAAGTGATGATCCGTAAAGT-3′, WlonR: 5′-GGCATAGAGAAAGTAAAAAGA-3′ (297780–298135 region), WmaeBF: 5′-CTGTGTGATAAGCAAGGAGT-3′, WmaeBR: 5′-TGGGTCAAATGGAGTAGGTA-3′ (469653–470116 region).

All samples were analysed for 2187C/T variants of *D. melanogaster* mtDNA by PCR with specific primers [32]. These variants correspond to the most ancient split in evolution of *D. melanogaster* mtDNA. In other words, they are markers of *M*- and *S*-clades [32]. The 343 bp mtDNA region (4586–4928 GenBank NC001709) of 143 samples was amplified with primers 04 and At6R [32] and sequenced to determine mitochondrial clades. Polymorphisms in this region allow the identification of III-, V-, VI-, I_II_III- and IV_VIII clades of mtDNA (Additional file 2). Samples of the *M*-mitotype (*n* = 111) were randomly chosen from both infected and uninfected flies with the addition of two samples harbouring wMel2. All available *S*-mitotype samples (*n* = 32) were analysed.

Amplicons were purified using a Zymoclean™ Gel DNA Recovery Kit (Zymo Research, USA) according to the manufacturer’s instructions, and sequenced by BigDye® Terminator v3.1 cycle sequencing kit (Applied Biosystems). Sequences were deposited in GenBank under accession numbers MG197842 – MG197984 for mtDNA analysis, and MG241453 – MG241491, MH010806 – MH010832 (Additional file 4).

All statistic calculations were performed in MS Excel (Microsoft Corporation) with the AtteStat 12.0.5 add-in.

## Results

### *Wolbachia* prevalence in Palearctic populations of *D. melanogaster*

To estimate *Wolbachia* prevalence, the 1251 *D. melanogaster* samples were examined. *Wolbachia* were found in all studied *D. melanogaster* Palearctic populations in the range of 0.33–1.0, with an average of 0.56 (Table 1; Additional file 5). The largest numbers of samples were collected from Kaliningrad, Crimea, Sakhalin localities and Nalchik city. In Kaliningrad Oblast, *Wolbachia* prevalence did not differ significantly (Fisher’s exact test, *P* = 0.06) over a two-year sampling period. There were also no differences in populations over four years in Nalchik including data from Bykov et al. [37] (Pearson’s chi-square, *P* = 0.35), or over a two-year period in Sakhalin (Fisher’s exact test, *P* = 0.82). The population of Izobilnoe (Crimea), which lives on the grape seed dump of the winery industry, had the greatest density of flies. The grape seed piles were swarmed with flies, and we assumed the population numbered at least hundreds of thousands of *D. melanogaster* individuals within a limited area. *Wolbachia* prevalence in this population was not different from the average prevalence rate of Palearctic populations (Fisher’s exact test, *P* = 0.55).

**Table 1:** Characteristic of 1505 *Drosophila melanogaster* samples collected in different Palearctic localities

Locality, year of collection	N	Prevalence (95% confidence interval)	*Wolbachia* genotypes of infected samples (rate)		*M*-mitotype rate in uninfected samples
			wMel	Other genotypes	
Western Europe					
Montpellier^1^, 2010	18	0.33 (0.13–0.59)	1.0	–	1.0
Northern Europe					
Gothenburg^2^, 2012	57	0.88 (0.76–0.95)	1.0	–	1.0
Central Europe					
Kaliningrad^3^, 2014**	234	0.51 (0.45–0.58)	1.0	–	1.0
Kaliningrad^3, 4^ 2015***	207	0.42 (0.35–0.49)	1.0	–	1.0
Eastern Europe					
Uman^5^, 2008	16	0.75 (0.48–0.93)	1.0	–	1.0
Uman^6^, 2012	12	0.42 (0.15–0.72)	1.0	–	1.0
Kiev^6^, 2012	13	0.85 (0.55–0.98)	1.0	–	1.0
Crimea, Alushta^6^, 2010	55	0.49 (0.35–0.63)	0.96	0.04 (a)	1.0
Crimea, Izobilnoe^6^, 2010	223	0.58 (0.52–0.65)	0.98	0.02 (a)	1.0
The Sinai Peninsula					
Sharm el-Sheikh^6^, 2010	24	0.87 (0.68–0.97)	0.67	0.33 (b)	1.0
North Caucasus					
Nalchik^2^, 2010	85	0.72* (0.61–0.81)	1.0*	–	0.96
Nalchik^2^, 2012	103	0.59* (0.49–0.69)	0.98*	0.02 (a)*	0.93
Nalchik^2^, 2013	66	0.67* (0.54–0.78)	0.98*	0.02 (a)*	1.0
Nalchik^2^, 2014	138	0.64 (0.56–0.72)	1.0	–	1.0
Central Asia					
Tashkent^7^, 2008	16	0.87 (0.62–0.98)	0.93	0.07 (c)	1.0
Western Siberia					
Novosibirsk^8^, 2008	57	0.67 (0.53–0.79)	1.0	–	1.0
Tomsk^9^, 2011	17	1.0 (0.80–1.0)	1.0	–	–
Biysk^6^, 2008	29	0.72 (0.53–0.87)	1.0	–	1.0
Cherga^6^, 2008	39	0.44 (0.28–0.60)	0.94	0.06 (c)	1.0
Iogach^8^, 2008	6	0.83 (0.36–0.99)	1.0	–	1.0
Far East					
Tomari^2^, 2014	28	0.43 (0.24–0.63)	1.0	–	0.88
Yuzhno-Sakhalinsk^2^, 2015	62	0.39 (0.27–0.52)	0.92	0.08 (d)	1.0

Notes: Samples were collected and provided by ^1^ – P.R. Haddrill; ^2^ – M.A. Voloshina; ^3^ – E.S. Melashchenko; ^4^ – M.A. Danilova; ^5^ – I.A. Kozeretska; ^6^ – Yu.Yu. Ilinsky; ^7^ – M.V. Zhukova; ^8^ – R.A. Bykov; ^9^ – Yu.M. Novikov. N – sample size. Prevalence given with 95% confidence intervals in parenthesis, estimated using the Clopper-Pearson method. Other *Wolbachia* genotypes: a – wMelCS2; b – wMel4; c – wMelCS; d – wMel2. * – Data were taken from Bykov et al. [37]. ** – Combined data from four localities of Kaliningrad city. *** – Combined data from eight localities of Kaliningrad city and Kaliningrad Oblast

**Fig. 1:**
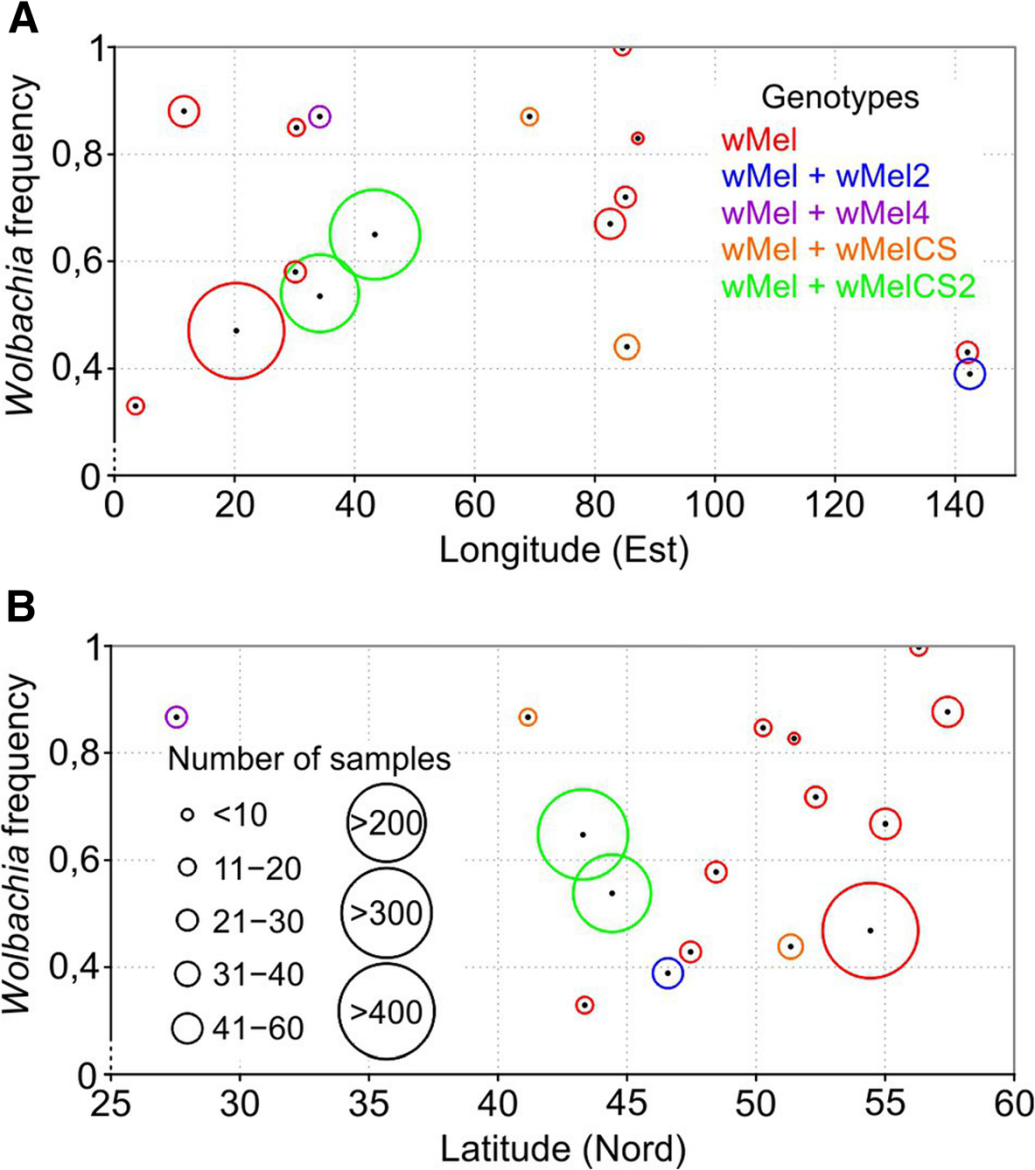
*Wolbachia* frequencies and genotype distribution in Palearctic populations of *D. melanogaster* by longitude (**a**) and latitude (**b**). For more information, see Additional file 6

Prevalence rates were compared in relation to latitude and longitude. Our data contained a latitude gap of 30°N-40°N and a longitude gap of 90°E-130°E. Therefore, the population locality positions of ~ 41°N-57°N and ~ 3°E-87°E were considered. As expected, Palearctic populations did not show any geographical pattern of *Wolbachia* prevalence (Fig. 1, Additional file 6), which is consistent with Kriesner et al. [45] but based on a larger sample size.

### *Wolbachia* genetic diversity in the populations

Five of six reported *Wolbachia* genotypes were found in Palearctic *D. melanogaster* populations (Table 1, Fig. 1). wMel was the only genotype in the majority of European localities and was predominant in other regions. It is noteworthy that the well-sampled Kaliningrad Oblast region had no cases of non-wMel genotypes, whereas other regions with a large sample size and some regions with small samples contained non-wMel variants, albeit at low frequencies. The high frequency of the wMel4 genotype detected in Sharm el-Sheikh may be explained by genetic drift in the small population. We also found two strains harbouring *Wolbachia* of the wMel2 genotype in one of two Far East localities. Two cases of wMelCS were found in the south of Western Siberia and Central Asia, wMelCS2 was found in the North Caucasus [37] and two localities of Crimea (Table 1, Fig. 1). Hence, our result confirms and expands upon previously reported data on Palearctic *D. melanogaster* populations [36, 46].

### CS genotype variation

*Wolbachia* variants of the CS group are rare in field collections but broadly distributed globally. Their genomes are very similar [30, 33, 38]. We checked for genetic differences among CS isolates of various origins. Three *Wolbachia* genome regions with parsimonious sites for every wMelCS and wMelCS2 sample were sequenced. Only two haplotypes were found (Table 2, Additional file 4), ‘CCG’ was common among wild-type and mutant stocks, and ‘CTG’ was found in only three samples of mutant stocks harbouring the wMelCS genotype. Therefore, we conclude that there is low genetic variation in *Wolbachia* of the CS group, which appears to contradict the idea of domination such variants in the past [29].

**Table 2:** Haplotypes of *Wolbachia* from CS-group genotypes. Origin, year of collection and genotype of fly stocks are indicated where available

Fly stock	*Wolbachia* genotype	*Wolbachia* haplotype (208.096, 297.946, 469.816)
w155, Central Asia, Uzbekistan, 1989 (wt)*	wMelCS2	CCG
w109, South-East Europe, Moldova, 1984 (wt)	wMelCS2	CCG
w115, Central Asia, Tajikistan, 1985 (wt)	wMelCS2	CCG
w181, Western Asia, Georgia, 1989 (wt)	wMelCS2	CCG
w214, Altai, 1992 (wt)	wMelCS2	CCG
w216, Altai, 1992 (wt)	wMelCS2	CCG
w238, Central Asia, Uzbekistan, 2005 (wt)	wMelCS2	CCG
AL42, Eastern Europe, Alushta, 2010 (wt)	wMelCS2	CCG
IZ-47, Eastern Europe, Izobilnoe, 2010 (wt)	wMelCS2	CCG
IZ-67, Eastern Europe, Izobilnoe, 2010 (wt)	wMelCS2	CCG
NL-12-1-5, North Caucasus, Nalchik, 2012 (wt)	wMelCS2	CCG
NL-35-13, North Caucasus, Nalchik, 2013 (wt)	wMelCS2	CCG
w153, Central Asia, Uzbekistan, 1989 (wt)	wMelCS	CCG
1–133, y^2^ cho^2^, 1981	wMelCS	CCG
3–1, ale, 1971	wMelCS	CCG
3–62, ve vn ri st, 1990s	wMelCS	CCG
3–64, vn st, 1988	wMelCS	CCG
w60b Canton-S	wMelCS	CCG
39, w; TM3, Sb / TM6, Tb, 2001	wMelCS	CCG
^1^w2, Portugal	wMelCS	CCG
^1^w6, Portugal	wMelCS	CCG
45, Cy/Sp; Sb Δ2–3 / TM6, 1995	wMelCS	CTG
1–128, y^596^z / TY;2 MR102, bw^v^, 1986	wMelCS	CTG
2–58, shr bw^2b^ abb sp. / SM5, 1995	wMelCS	CTG
^2^Canton-S, 1930	wMelCS	CTG
^2^VF-0058-3	wMelCS	CTG
^2^Popcorn/w^1118^	wMelPop	CTG
^2^Kurdamir, Azerbaijan, 1977	wMelCS2	CCT
^2^Anapa-79, Russia, Anapa, 1979	wMelCS2	CCT
^3^DGRP335, USA, 2008	?	TCG
^3^DGRP338, USA, 2008	?	TCG

^1^Versache et al. [38]; ^2^Chrostek et al. [30]; ^3^Richardson et al. [33]; *wt – wild-type stock

### mtDNA variants in the populations

Samples of mtDNA from infected and uninfected *D. melanogaster* populations were tested for 2187C/T variants*.* The purpose of this analysis was *i*) to check any facts of mtDNA/*Wolbachia* coinheritance disorders, which would indicate horizontal transmission of *Wolbachia* or mtDNA paternal passing, and *ii*) to compare M/S ratios in infected vs. uninfected flies, which would indirectly indicate the origin of uninfected flies.

No mtDNA/*Wolbachia* coinheritance disorders were revealed. All samples infected with *Wolbachia* of the MEL genotype were *M*-mitotype, and all samples from the CS group were *S*-mitotype. In uninfected samples, the *M*-mitotype was found in all localities, whereas the *S*-mitotype was only found in North Caucasus and the Far East (Table 1). No significant difference was revealed in *M/S* ratios of infected vs. uninfected lines (Fisher’s exact test, *P* = 0.114), which statistically indicates the recent loss of the infection by ancestors of *S*-mitotype uninfected flies. These results confirm the previous data of Ilinsky [32].

To characterize the mitotype diversity in detail, the 343 bp mtDNA region was sequenced for 143 samples. Among *M*-mitotype samples of the I_II_III group, III and V clades were found in similar proportion across the studied Palearctic territory (Table 3). The proportion, in particular, I_II_III group + III clade (‘III+’ clade) vs. V clade was not significantly different (Fisher’s exact; *P* = 0.55). There was no difference in infection prevalence in ‘III+’ vs. the V clade (Fisher’s exact; *P* = 1.0). Two samples with IV_VIII clades (both wMel2-infected), were found only in the Far East population. All *S*-mitotype samples from Eastern Europe to the Far East were confirmed to have the canonical VI-clade sequence.

**Table 3:** The mtDNA clades of 143 *D. melanogaster* samples from 12 regions of Palearctic

Region	mtDNA clade (infected/uninfected samples)				
	*M*-clade				*S*-clade^a^
	I_II_III	III	V	IV_VIII	VI
Western Europe	1/−	–	1/1	–	–
Northern Europe	1/−	3/2	–	–	–
Central Europe	5/1	3/1	3/2	–	–
South-East Europe	–	–	1/−	–	1/−
Eastern Europe	1/3	6/4	14/12	–	1/3
The Sinai Peninsula	1/1	2/1	–	–	–
North Caucasus	–	1/1	4/3	–	2/6
Western Asia	–	–	−/1	–	1/1
Central Asia	3/−	−/1	6/2	–	4/3
Ural	–	–	2/−	–	–
Altai	–	1/−	4/−	–	2/1
Far East	2/3	2/2	1/−	2/−	−/7
Total:	14/8	18/12	36/21	2/−	11/21

^a^All available samples with *S*-mitotype were used in analysis

## Discussion

Here, the mtDNA and *Wolbachia* endosymbiont of *D. melanogaster* were examined in population and phylogeographic terms across the vast Palearctic territory. Our data are consistent with previous observations on *i*) widespread *Wolbachia* infection in *D. melanogaster* populations [29, 35, 47], *ii*) predominance of the wMel genotype [29, 35], and *iii*) strict coinheritance of *Wolbachia* and mtDNA variants [32–34, 36, 48]. We demonstrate that fly populations of the temperate zone renew after a cold season. No changes were observed in diversity and rate of maternal factors in *D. melanogaster* populations of Central (Kaliningrad) and Eastern Europe (Nalchik), and Western Siberia (Altai). No geographical pattern of *Wolbachia* infection rate was observed in the Palearctic, which corresponds well with the results of Kriesner et al. [45]. However, rare *Wolbachia* variants were found in certain regions, viz. wMelCS2 from Western Siberia to Eastern Europe [29, 35, 36], wMel2 in the Far East: China, Japan [30, 34], and Sakhalin; wMel4 in Sinai peninsula [32].

In the present survey, *Wolbachia* variants of the CS group were identified. The wMelCS2 genotype was detected in Eastern Europe and North Caucasus, and two samples with wMelCS infection were found in Western Siberia and Central Asia. Riegler et al. [29], proposed a hypothesis of wMelCS replacement by wMel in the XX century based on the observation that wMelCS is primarily present in populations before 1970 and further the wMel genotype become dominant. If that is the case, high genetic diversity for CS *Wolbachia* group and *S*-clade mitochondrial DNA should be observed, whereas diversity of the MEL group and *M*-clade mtDNA should be rather low. In fact, we observed the opposite situation. There were several lineages within the MEL group and *M*-clade mtDNA, and only one lineage for the CS group and *S*-clade mtDNA [30, 33]. Here, we tried to reveal genetic differentiation of CS genotypes using three SNPs located in different protein-coding genes. It is obvious the ‘CCG’ haplotype is an ancestral, as it is shared by both MEL and CS *Wolbachia* groups. Moreover, the ‘CCG’ haplotype was found among wMelCS and wMelCS2 genotypes, and other haplotypes seem to be local variants, namely ‘TCG’ in North America and ‘CCT’ in South-East Asia. Several isolates of the ‘CTG’ haplotype, which are found in wMelCS-infected stocks, could be the result of using one or more sources of maternal laboratory stock(s). Thus, we observed low genetic variation within the CS lineage of *Wolbachia,* which, together with above-mentioned comparison of MEL/*M* and CS/*S* diversity, and low diversity of the VI mtDNA lineage contradicts the hypothesis of recent replacement of *Wolbachia* genotypes. It is difficult to imagine different mitotypes supplanting the VI clade variants. The reason for the high proportion of wMelCS genotypes in populations before 1970 could be a case of sample error. Indeed, the number of stocks established with flies collected before the 1980s is very low (Additional file 5). This inference should be confirmed by more detailed analyses of both *Wolbachia* and mtDNA. An alternative scenario that cannot be ruled out is an increase in the wMelCS genotype rate in *D. melanogaster* populations during the first part of the XX century or earlier. This increasing could be due to specific interactions between wMelCS *Wolbachia* genotypes with unknown factors, the latter could be sigma virus [49, 50] or P-element [51, 52] that have recently invade *D. melanogaster* populations.

Coinheritance of *Wolbachia* variants and host mtDNA haplotypes has been reported for different species [1, 7, 21, 53–58]. The association between *D. melanogaster* mtDNA and *Wolbachia* genotypes has also been demonstrated in several studies [32–34]. Our *M* and *S* mtDNA clades distribution data in Palearctic *D. melanogaster* populations and their coinheritance with *Wolbachia* genotypes confirms the strict association between symbiont and mitochondrial lineages. Two main *D. melanogaster* mtDNA lineages (clades) within the *M*-clade were revealed. The ‘III+’ lineage consists of clades I and III, which are widespread all over the world [30, 32–34, 38], and clade II, which was found only in African *D. melanogaster* populations [33, 34]. Some samples of the ‘III+’ clade were identified as clade III by 4616A/T (Additional file 1). Therefore, we cannot exclude that samples with the 4616(A) substitution could also be clade III, and further analysis is required. The V clade of mtDNA was previously reported in *D. melanogaster* populations from Western Europe [33, 34, 38], Central and North Asia [32]. In the present study, this clade was also found in the Far East. Thus, we can assume that the V mtDNA clade is common for Palearctic *D. melanogaster* populations. *D. melanogaster* with the V clade of mtDNA were previously shown to be more viable in cold conditions than other clades [38], that could explain high frequency of this clade in Palearctic. However, the question of a cold tolerance mechanism determined by mtDNA remains unclear. The same rate of *Wolbachia* infection within *D. melanogaster* with ‘III+’ and V mtDNA clades may indicate a recent loss of the symbiont.

## Conclusions

Our in-depth survey of maternal-inherited factors of Palearctic *D. melanogaster* populations is consistent with previous studies and expands our knowledge. Prevalence of *Wolbachia* infection does not have a specific distribution pattern in the Palearctic. The wMel genotype inhabits every population, whereas other genotypes are rare and localised. Variants of V and ‘III+’ mitochondrial clades predominate in infected and uninfected flies across the Palearctic territory. According to symbiont and mtDNA diversity, the fly populations of many regions in temperate zones renew after the cold season, and the contribution of fly migration is not detected. Low genetic polymorphism of CS genotypes together with mitotypes and *Wolbachia* infection of global *D. melanogaster* populations do not support the hypothesis of a recent global *Wolbachia* genotype replacement. However, an increase in the wMelCS genotype rate in global *D. melanogaster* populations due to interactions with specific factors cannot be excluded.

## Additional files

Additional file 1: List of *Drosophila melanogaster* stocks used for analysis of 343 bp mtDNA region. GenBank numbers of sequencing samples are indicated. (XLS 55 kb)

Additional file 2: Polymorphic sites of 343 bp mtDNA region of *Drosophila melanogaster* (according to GenBank NC001709). (XLS 37 kb)

Additional file 3: Haplotypes of *Wolbachia* from CS-group genotypes. GenBank accession numbers are indicated in brackets where available. (DOC 74 kb)

Additional file 4: Nucleotide polymorphisms of CS *Wolbachia* variants. Positions chosen for analysis performed in the current study are highlighted in yellow. Uncertain nucleotides highlighted in grey. (XLS 43 kb)

Additional file 5: Dataset of *Wolbachia* genotypes found in worldwide populations of *Drosophila melanogaster* during the 1925–2015 period. (XLS 72 kb)

Additional file 6: Dataset used for analysis of geographical *Wolbachia* patterns. (XLS 31 kb)
