# Supplementary material for: Prevalence and genetic diversity of Wolbachia endosymbiont and mtDNA in Palearctic populations of Drosophila melanogaster
Source: BMC Evol Biol. 2019 Feb 26;19(Suppl 1):48. doi: 10.1186/s12862-019-1372-9 (PMC6391860; doi:10.1186/s12862-019-1372-9)
Supplement: Supplementary file 3 — Haplotypes of Wolbachia from CS-group genotypes. GenBank accession numbers are indicated in brackets where available. (DOC 74 kb) [file 12862_2019_1372_MOESM3_ESM.doc]

Additional file 3. Haplotypes of *Wolbachia* from CS-group genotypes. GenBank accession numbers are indicated in brackets where available.

| **Fly stock, origin, (reference)** | ***Wolbachia* Genotype** | **Position in *Wolbachia* genome**  **(according to GenBank AE017196.1)** | | |
| --- | --- | --- | --- | --- |
|  |  | **208.096** | **297.946** | **469.816** |
| DGRP335, USA, 2008, (Richardson et al. [33]) | ? | T | C | G |
| DGRP338, USA, 2008, (Richardson et al. [33]) | ? | T | C | G |
| Kurdamir, Azerbaijan, 1977, (Chrostek et al. [30]) | wMelCS2 | C | C | T |
| Anapa-79, Russia, Anapa, 1979, (Chrostek et al. [30]) | wMelCS2 | C | C | T |
| w155, Central Asia, Uzbekistan, 1989 | wMelCS2 | C  (MG241454) | C  (MG241467) | G  (MG241480) |
| w109, South-East Europe, Moldova, 1984 | wMelCS2 | C  (MG241453) | C  (MG241466) | G  (MG241479) |
| w115, Central Asia, Tajikistan, 1985 | wMelCS2 | C  (MG241454) | C  (MG241467) | G  (MG241480) |
| w181, Western Asia, Georgia, 1989 | wMelCS2 | C  (MG241464) | C  (MG241477) | G  (MG241490) |
| w214, Altai, 1992 | wMelCS2 | C  (MG241456) | C  (MG241469) | G  (MG241482) |
| w216, Altai, 1992 | wMelCS2 | C  (MG241457) | C  (MG241470) | G  (MG241483) |
| w238, Central Asia, Uzbekistan, 2005 | wMelCS2 | C  (MG241458) | C  (MG241471) | G  (MG241484) |
| AL42, Eastern Europe, Alushta, 2010 | wMelCS2 | C  (MG241461) | C  (MG241474) | G  (MG241487) |
| IZ-47, Eastern Europe, Izobilnoe, 2010 | wMelCS2 | C  (MG241462) | C  (MG241475) | G  (MG241488) |
| IZ-67, Eastern Europe, Izobilnoe, 2010 | wMelCS2 | C  (MG241463) | C  (MG241476) | G  (MG241489) |
| NL-12-1-5, North Caucasus, Nalchik, 2012 | wMelCS2 | C  (MG241459) | C  (MG241472) | G  (MG241485) |
| NL-35-13, North Caucasus, Nalchik, 2013 | wMelCS2 | C  (MG241460) | C  (MG241473) | G  (MG241486) |
| Canton-S, 1930, (Chrostek et al. [30]) | wMelCS | C | T | G |
| VF-0058-3, (Chrostek et al. [30]) | wMelCS | C | T | G |
| Popcorn/w^1118^, (Chrostek et al. [30]) | wMelPop | C | T | G |
| w2, Portugal, (Versache et al. [38]) | wMelCS | C | C | G |
| w6, Portugal, (Versache et al. [38]) | wMelCS | C | C | G |
| w153, Central Asia, Uzbekistan, 1989 | wMelCS | C  (MG241465) | C  (MG241478) | G  (MG241491) |
| 1-128, y^596^z / TY;2 MR102, bw^v^, 1986 | wMelCS | C  (MH010815) | T  (MH010806) | G  (MH010824) |
| 1-133, y^2^ cho^2^, 1981 | wMelCS | C  (MH010816) | C  (MH010807) | G  (MH010825) |
| 2-58, shr bw^2b^ abb sp / SM5, 1995, (Umea DSC №51700 - №101556, 2001y. Kyoto) | wMelCS | C  (MH010817) | T  (MH010808) | G  (MH010826) |
| 3-1, ale, 1971 | wMelCS | C  (MH010818) | C  (MH010809) | G  (MH010827) |
| 3-62, ve vn ri st, 1990s | wMelCS | C  (MH010819) | C  (MH010810) | G  (MH010828) |
| 3-64, vn st, 1988 | wMelCS | C  (MH010820) | C  (MH010811) | G  (MH010829) |
| 39, w; TM3, Sb / TM6, Tb, 2001 | wMelCS | C  (MH010821) | C  (MH010812) | G  (MH010830) |
| 45, Cy/Sp; Sb Δ2-3 / TM6, 1995 | wMelCS | C  (MH010822) | T  (MH010813) | G  (MH010831) |
| w60b (Canton-S) | wMelCS | C  (MH010823) | C  (MH010814) | G  (MH010832) |
